# Two-year longitudinal change in choroidal and retinal thickness in school-aged myopic children: exploratory analysis of clinical trials for myopia progression

**DOI:** 10.1186/s40662-022-00276-4

**Published:** 2022-02-01

**Authors:** Meiping Xu, Xinping Yu, Minghui Wan, Kemi Feng, Junxiao Zhang, Meixiao Shen, Björn Drobe, Hao Chen, Jia Qu, Jinhua Bao

**Affiliations:** 1 Eye Hospital and School of Ophthalmology and Optometry, Wenzhou Medical University, Wenzhou, Zhejiang China; 2 National Clinical Research Center for Ocular Diseases, Wenzhou, Zhejiang China; 3 WEIRC, WMU-Essilor International Research Centre, Wenzhou, Zhejiang China; 4 R&D Vision Sciences AMERA, Essilor International, Singapore, Singapore

**Keywords:** Axial length, Choroidal thickness, Children, Myopia, Spectral-domain optical coherence tomography

## Abstract

**Background:**

With increasing axial length and myopia progression, the micro-structure of the retina and choroid gradually changes. Our study describes the longitudinal changes in retinal and choroidal thickness in school-aged children with myopia and explores the relationship between changes in choroidal thickness and myopia progression.

**Methods:**

An exploratory analysis of a randomized trial was performed. Children (n = 168, aged 7 to 12 years) with myopia from − 0.75 dioptre (D) to − 4.00 D were enrolled in this prospective longitudinal study. Cycloplegic refraction, axial length (AL), retinal and choroidal thicknesses were measured at baseline and at 1- and 2-year follow-ups. “Rapid progression myopia” was defined as increasing in myopia > 1.00 D and “stable progression myopia” was ≤ 1.00 D during the 2-year follow-up. Factors affecting the changes in choroidal thickness were analysed using linear mixed models.

**Results:**

AL significantly increased by 0.67 ± 0.24 mm with a myopic shift of − 1.50 ± 0.64 D over the 2 years. The overall retinal thickness increased from 251.12 ± 15.91 µm at baseline to 253.47 ± 15.74 µm at the 2-year follow-up (F = 23.785, *P* < 0.001). The subfoveal choroidal thickness decreased from 231.03 ± 54.04 µm at baseline to 206.53 ± 59.71 µm at the 2-year follow-up (F = 73.358, *P* < 0.001). Choroidal thinning was significantly associated with AL elongation (β =  − 43.579 μm/mm, *P* = 0.002) and sex (β =  − 17.258, *P* = 0.001). Choroidal thickness continued to decrease in subjects with rapid progression (F = 92.06, *P* < 0.001) but not in those with steady progression (F = 2.23, *P* = 0.119).

**Conclusion:**

Significant choroidal thinning was observed and was associated with rapid progression and sex. These findings indicate a need to understand the role of the choroid in eye growth and myopia development.

**Synopsis/Precis:**

The macular choroidal thickness of myopic children is relevant to different degrees of myopic progression in this 2-year longitudinal study. These findings suggest that control of choroidal thickness might work to regulate human ocular growth.

*Trial registration* Chinese Clinical Trial Register (ChiCTR): ChiCTR-INR-16007722

**Supplementary Information:**

The online version contains supplementary material available at 10.1186/s40662-022-00276-4.

## Background

Myopia is widely perceived as a global issue, with the incidence rate increasing year by year, especially in Asian countries [1–3]. While the underlying pathogenesis of myopic development is still elusive, the effectiveness of interventions for myopia prevention and control is limited. Previous animal experimental studies [4–6] confirmed that the choroid might play an important role in refractive adjustment and eye growth. The eye can detect retinal myopic defocus (image-focused in front of the retina), or hyperopic defocus (image-focused behind the retina) and undergo compensatory growth by increasing or decreasing choroidal thickness (CT). Thus, CT changes precede axial length changes and scleral remodelling [5, 7]. The change in subfoveal CT could be related to changes in choroidal blood flow [8, 9], lymphatics [10], nonvascular smooth muscle [11] and choroidal vascularity index [12, 13]. Zhou and colleagues found that changes in CT were positively correlated with changes in choroidal blood flow in guinea pig myopia [14] and increased choroidal blood flow attenuates scleral hypoxia as well as inhibits myopia development [15]. In addition, clinical studies also found significantly decreased CT and choroidal blood perfusion after near work, which is a risk factor for myopia [16–18]. Such decreased choroidal blood perfusion might cause scleral hypoxia.

Morphological analysis of the fundic retina and choroid depends on acquiring clear optical coherence tomography (OCT) images and segmentation of the posterior segment layers either manually or by computer programs that automatically or semi-automatically identify the layer interfaces. Previous studies have revealed the clinical characteristics of subfoveal CT and associated factors such as age, sex, birth parameters, puberty, ethnicity, refractive dioptre (D), and axial length (AL) [19–22]. Some cross-sectional studies have found that CT may increase during childhood and become the thickest during adolescence [20, 23, 24]. CT has been shown to be thinner in myopes than in hyperopes and even thinner in high myopes [25, 26]. There are relatively few longitudinal studies, even then their conclusions are somewhat controversial. Hansen et al. confirmed that the CT of children aged 11 to 16 increased, while the increase was greater in hyperopes and lesser in myopes [27]. Jin et al. demonstrated CT reduction in those who developed myopic shift [28]. Xiong et al. found a marked decrease in CT in patients with newly developed myopia but not in those with persistent myopia [29]. Such divergent findings may be related to ethnic differences, different age ranges, and different refractive power changes.

Recent studies have suggested that choroidal thinning occurs in the early period of myopic development [29, 30], but age-related changes in myopic choroidal thinning and the association with myopic progression have not been established quantitatively. The main purpose of this 2-year longitudinal study was to observe the nature and time course of changes in CT in children aged 7 to 12 years with low-to-moderate myopia (− 0.75 to − 4.00 D). A second goal was to explore factors that affect the changes in CT in myopic children as well as the potential role of the choroid in myopia progression.

## Materials and methods

### Setting and participants

This prospective, longitudinal study was conducted according to the tenets of the Declaration of Helsinki. It was approved by the Institutional Review Board of the Eye Hospital of Wenzhou Medical University. After the participants understood the study protocol and possible consequences of the study, consent was obtained from the participating children and their parents or other guardians.

This study was a part of the Personalized Addition Lenses Clinical Trials (PACT) [31], which was a randomized clinical trial to determine if myopia progression is different between children wearing personalized progressive addition lenses (PPALs) versus + 2.00 D fixed progressive addition lenses (FPALs) or single vision lenses (SVLs). The study was registered in the Chinese Clinical Trial Register (ChiCTR, ChiCTR-INR-16007722). The inclusion criteria were as follows: children with age 7 to 12 years and spherical equivalent of refraction (SER) between − 4.00 and − 0.75 D as measured by cycloplegic autorefraction in both eyes. The participants were excluded for the following reasons: best-corrected visual acuity (BCVA) less than 20/25, anisometropia > 1.0 D in SER, history of intraocular surgery, or ocular comorbidities, including strabismus.

### Research methods

The children (n = 210) were enrolled from the Optometry Clinic of the Eye Hospital of Wenzhou Medical University from July 1, 2014, to February 1, 2015. Essential information included sex, age, time of occurrence of myopia, birth history, and refractive status of their parents. Each child was randomly assigned to wear either PPALs, FPALs, or SVLs.

The enrolled participants were followed up every 6 months for 2 years, and underwent comprehensive ophthalmic examinations, including BCVA, cycloplegic refraction, anterior segment, fundus examination [including spectral-domain optical coherence tomography (SD-OCT) to measure the thickness of the choroid and retina], and AL. These tests were performed at each follow-up visit, except for SD-OCT, which was performed once a year. After corneal anaesthesia was induced with one drop of proparacaine (0.5% Alcaine, Alcon Laboratories, Ft. Worth, TX, USA), cycloplegia was achieved by administering three drops of 1% cyclopentolate (Alcon Laboratories) 5 min apart. Five consecutive and reliable autorefraction (Canon RK-F1; Canon Inc., Tokyo, Japan) measurements were obtained 30 min after the third drop was administered. There were conflicting reports regarding the influence of cyclopentolate on the AL and CT [32–35]. To obtain high quality SD-OCT images and consistent results, the AL and SD-OCT were measured after cycloplegic autorefraction in this study. SER was used to classify the refractive status. AL was measured by a Lenstar LS900 (Haag-Streit AG, Koeniz, Switzerland).

### SD-OCT procedure and data collection

**Fig. 1:**
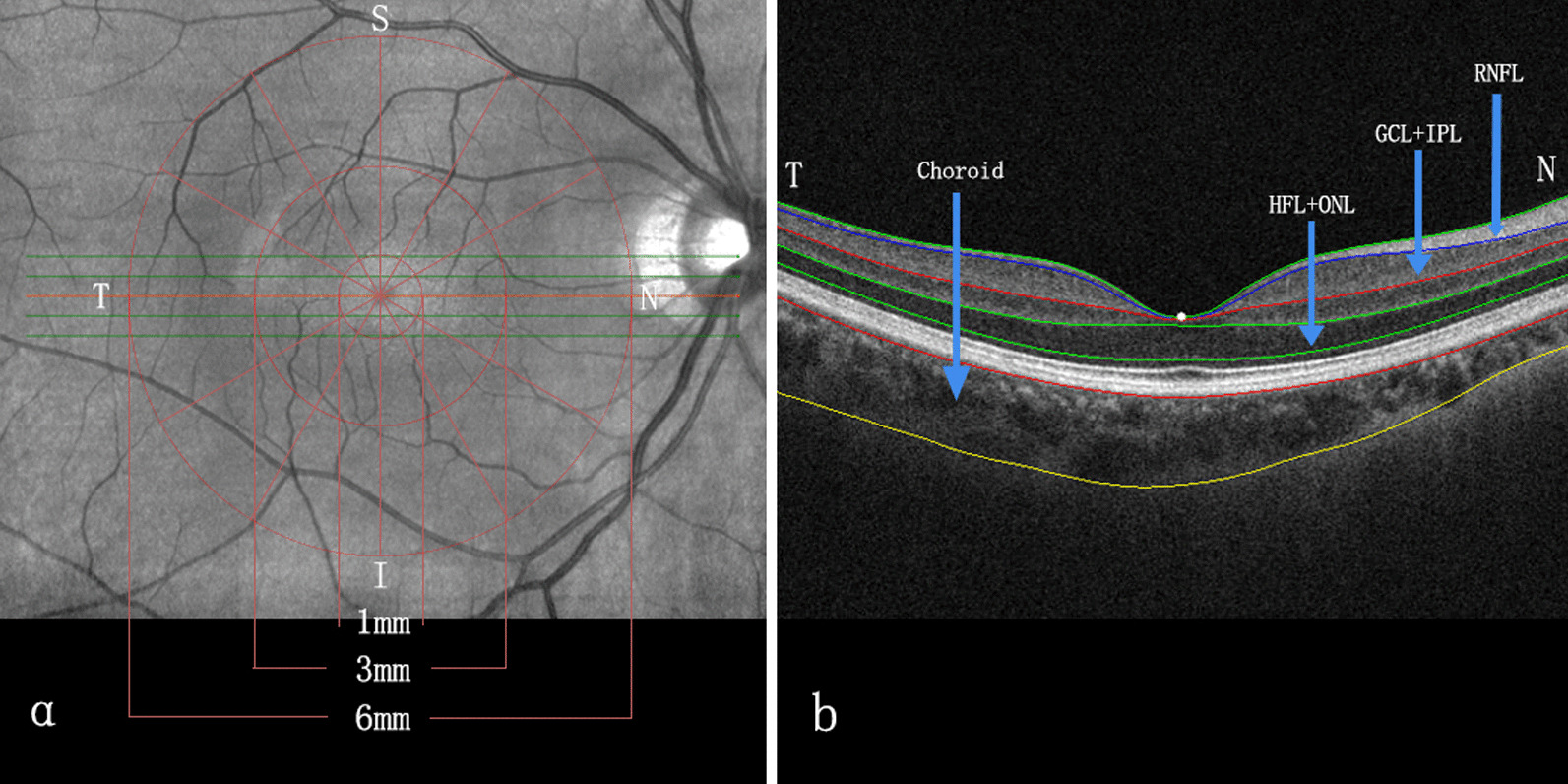
Cross-sectional and en face segmentation of choroidal and retinal thickness measurements obtained by SD-OCT. **a** The macula was divided into nine parts: the central fovea and the temporal, nasal, inferior, and superior regions of the parafovea and perifovea. The diameter of the central foveal region was 1 mm. The parafoveal region ranged from 1 to 3 mm from the central foveal region, and the perifoveal region ranged from 3 to 6 mm from the central foveal region. **b** The boundaries of the fundus structure were segmented by an automated algorithm, and the thickness profiles of the macular outer retinal sub-layers and choroid were determined. S, superior; N, nasal; I, inferior; T, temporal; HFL + ONL, Henle fibre layer and outer nuclear layer; GCL + IPL, ganglion cell layer and inner plexiform layer; RNFL, retinal nerve fibre layer

All participants underwent a macular scan using the Cirrus 4000 HD-OCT (Zeiss, Oberkochen, Germany) with the macular radial 512 × 128 cube scan pattern. The HD 5 Line Raster modality was selected, and the rotation was adjusted for 30° intervals. Finally, a series of six radial SD-OCT scans, each separated by 30° and centred on the fovea, were performed. Each raster scan covered a nominal 6-mm square grid area centred on the fovea (Fig. 1). SD-OCT scans were performed between 3:00 and 7:00 p.m. to limit the potential confounding influence of diurnal variations in CT [36, 37]. All SD-OCT scans were reviewed for image quality, with a signal strength indicator value ≥ 7. Images were excluded under the following conditions: signal strength less than 7, motion artefact, involuntary saccade, obvious decentration misalignment, or algorithm segmentation failure due to unclear boundaries between the retina layers.

Three concentric circles centred on the fovea were applied once the tomography map was obtained. This divided the macula into three sub-fields: the central foveal zone (diameter = 1 mm), the parafoveal zone (diameter = 3 mm), and the perifoveal zone (diameter = 6 mm). The radial scan divided the three concentric circles into nine parts, i.e., the central fovea and the temporal, nasal, inferior, and superior regions of the parafovea and the perifovea (Fig. 1).

Following image acquisition at each follow-up visit, all SD-OCT images exported from the instrument were analysed using custom-written software [38]. The software included correction of the image magnification based on the refractive power and was used to quantify the thickness of the fundic retinal sub-layers and the choroid, as previously described [39–41]. The standards for the segmentation of each layer were defined according to the International Nomenclature for Optical Coherence Tomography Panel [42]. The average regional thicknesses of the retinal nerve fibre layer (RNFL), ganglion cell layer and the inner plexiform layer (GCL + IPL), Henle fibre layer and outer nuclear layer (HFL + ONL), total retina, and choroid of each sub-field were measured by custom-developed software and used to analyse the changes at the 1- and 2-year follow-up visits. An experienced retinal specialist analysed all the images. If automated segmentation errors occurred or resulted in measurement artefacts, manual segmentation was performed.

### Statistical analysis

Statistical analysis was performed using SPSS software version 20.0 (IBM Corp., Armonk, NY, USA). Data for only the right eyes were included for statistical analysis. SER values were calculated as the sphere power + (cylinder power/2). Changes in CT in the different macular regions were calculated as the CT at baseline minus the CT at the 2-year follow-up examination. We defined “rapid progression myopia” as increases in myopia > 1.00 D during the 2-year follow-up, “stable progression myopia” was defined as increases ≤ 1.00 D during the 2-year follow-up according to a mean progression of − 1.20 D over 2 years in a clinical study [43].

The parameters were calculated as the means ± standard deviations for the continuous variables and rates (proportions) for the categorical variables. One-way analysis of variance (ANOVA) was used to test for differences among the PPAL, FPAL, and SVL groups. Repeated measures analysis of variance (RM-ANOVA) was used to test the parameters for differences among the baseline, 1-year follow-up, and 2-year follow-up visits. For multiple measurements, Bonferroni correction was applied for pairwise comparisons. The intergroup differences were tested with independent sample t-tests between sexes and between the 7- to 9-year-old and the 10- to 12-year-old participants. The relationship between the changes in CT and AL was tested by linear correlation. Factors associated with longitudinal changes in CT were analysed by multiple regression analysis.

## Results

### Patient characteristics

Of the initial 210 patients enrolled, 30 were lost to follow-up or had incomplete data, and 12 were excluded due to poor image quality. For the remaining 168 patients, the mean age at baseline was 9.3 ± 1.1 years (range, 7 to 12 years), 52.4% of whom were males (Table 1). The baseline SER and AL values of the right eyes were − 2.38 ± 0.61 D (range, − 0.75 to − 3.75 D) and 24.58 ± 0.72 mm (range, 22.82 to 26.85 mm), respectively. At the 2-year follow-up examination, the changes in the SER and AL values for the PPAL, FPAL, and SVL groups were − 1.42 ± 0.69 D and 0.65 ± 0.26 mm, − 1.48 ± 0.57 D and 0.65 ± 0.23 mm, and − 1.61 ± 0.64 D and 0.70 ± 0.23 mm, respectively. There were no significant differences among the groups (F = 0.625, *P* = 0.537 and F = 0.041, *P* = 0.960). The baseline subfoveal CT values for the PPAL, FPAL, and SVL groups were 231.60 ± 51.84 µm, 230.46 ± 57.36 µm, and 231.65 ± 56.77 µm, respectively. After 2 years of follow-up, the CT values for these groups were 202.17 ± 56.7793 µm, 210.87 ± 68.29 µm, and 209.51 ± 64.98 µm, respectively. Changes in CT during the first and second year were not significantly different among the three groups (F = 1.888, *P* = 0.155 and F = 0.550, *P* = 0.578, for the first and second year separately.)

**Table 1:** Baseline data characteristics of the 168 participants and comparison among the PPAL, FPAL, and SVL groups

Parameter^a^	Total (*n* = 168)	PPALs (*n* = 59)	FPALs (*n* = 48)	SVLs (*n* = 61)	*P*
Age (years)	9.3 ± 1.1	9.2 ± 1.1	9.4 ± 1.1	9.2 ± 1.1	0.472
Male, n (%)	88 (52.4%)	32 (54.2%)	23 (47.9%)	33 (54.1%)	0.849
SER (D)	− 2.38 ± 0.61	− 2.41 ± 0.68	− 2.34 ± 0.56	− 2.39 ± 0.57	0.852
VA (logMAR)	0.00 ± 0.02	0.00 ± 0.02	− 0.01 ± 0.02	− 0.01 ± 0.03	0.222
AL (mm)	24.58 ± 0.72	24.55 ± 0.71	24.58 ± 0.78	24.61 ± 0.69	0.900
Subfoveal CT (µm)	231.03 ± 54.04	231.60 ± 51.84	230.46 ± 57.36	231.65 ± 56.77	0.981
RT (µm)	251.12 ± 15.91	250.58 ± 15.15	252.31 ± 17.11	251.18 ± 15.73	0.853
ΔSER1 (D)	− 1.02 ± 0.43	− 1.08 ± 0.42	− 1.00 ± 0.37	− 0.99 ± 0.48	0.376
ΔSER2 (D)	− 0.47 ± 0.38	− 0.47 ± 0.36	− 0.48 ± 0.37	− 0.45 ± 0.40	0.923
ΔAL1 (mm)	0.36 ± 0.15	0.38 ± 0.14	0.35 ± 0.13	0.36 ± 0.17	0.688
ΔAL2 (mm)	0.29 ± 0.13	0.29 ± 0.12	0.29 ± 0.13	0.30 ± 0.13	0.936
ΔCT1 (µm)	− 7.98 ± 23.61	− 12.01 ± 25.97	− 8.15 ± 22.06	− 3.67 ± 21.85	0.155
ΔCT2 (µm)	− 16.64 ± 25.631	− 17.42 ± 26.45	− 13.43 ± 27.03	− 18.46 ± 23.72	0.578

*PPALs=* personalized progressive addition lenses; *FPALs=* fixed progressive addition lenses; *SVLs=* single vision lenses; *n=* number; *D=* dioptre; *SER=* spherical equivalent of refraction; *logMAR=* logarithm of the minimum angle of resolution; *VA=* visual acuity; *AL=* axial length; *CT=* choroidal thickness; *RT=* retinal thickness

^a^The means ± standard deviations for age, SER, VA, and AL; *P* values based on ANOVA. ΔSER1, the change of spherical equivalent of refraction in the first year; ΔSER2, the change of spherical equivalent of refraction in the second year; ΔAL1, the change of axial length in the first year; ΔAL2, the change of axial length in the second year; ΔCT1, the change of subfoveal choroidal thickness in the first year; ΔCT2, the change of subfoveal choroidal thickness in the second year

### Changes in SER, AL, and retinal and choroidal thickness

Since there were no significant differences in the changes in SER, AL, or CT values among the groups at the 2-year follow-up visit, the following data were analysed for all 168 patients. During the study period, the SER and AL values increased significantly, from − 2.39 ± 0.61 D and 24.58 ± 0.72 mm at baseline to − 3.88 ± 0.92 D and 25.24 ± 0.76 mm at the 2-year follow-up (*P* < 0.001, Table 2). Furthermore, there were significant correlations between the changes in AL and the changes in SER (first year, r =  − 0.661, B =  − 0.234 and second year r =  − 0.677, B =  − 0.233; both *P* < 0.001).

**Table 2:** Longitudinal parameters of 168 participants at baseline and follow-up visits

Parameter^c,d^	Baseline	One-year follow-up	Two-year follow-up	ΔThickness	F	*P*
SER (D)	− 2.39 (− 2.75, − 2.00)	− 3.41 (− 4.00, − 2.87)	− 3.88 (− 4.50, − 3.25)	–	798.16	< 0.001
AL (mm)	24.58 (24.04, 25.08)	24.94 (24.43, 25.44)	25.24 (24.71, 25.71)	–	1113.87	< 0.001
Retina (µm)	251.12 ± 15.91^a^	251.09 ± 16.34^b^	253.47 ± 15.74^a,b^	–	23.785	< 0.001
RNFL (µm)	14.47 ± 1.96^a^	14.93 ± 2.23^a,b^	14.31 ± 2.31^a^	–	3.652	0.027
GCL + IPL (µm)	24.34 ± 5.96	24.31 ± 5.85	24.63 ± 6.07	–	1.407	0.352
HFL + ONL (µm)	88.29 ± 10.67^a,b^	90.74 ± 10.92^a^	90.16 ± 10.49^b^	–	10.262	< 0.001
Choroid (µm)						
Central	231.03 ± 54.04	223.71 ± 56.76	206.53 ± 59.71	24.63 ± 31.16	66.635	< 0.001
Para-N	197.44 ± 55.73	190.25 ± 57.16	175.36 ± 61.67	22.08 ± 26.61	81.990	< 0.001
Para-T	241.78 ± 53.88	229.81 ± 53.11	216.47 ± 60.57	24.30 ± 33.89	58.673	< 0.001
Para-S	233.09 ± 50.10	228.39 ± 54.66	214.97 ± 56.65	18.11 ± 34.06	32.074	< 0.001
Para-I	236.17 ± 54.42	228.91 ± 58.02	212.57 ± 61.23	23.59 ± 32.39	59.813	< 0.001
Peri-N	155.47 ± 49.74	152.75 ± 50.89	139.12 ± 52.79	16.35 ± 22.65	61.377	< 0.001
Peri-T	244.04 ± 53.80	234.45 ± 52.27	221.23 ± 60.11	22.81 ± 38.93	38.841	< 0.001
Peri-S	232.69 ± 51.47	231.60 ± 52.17	221.48 ± 56.13	11.20 ± 38.41	11.048	< 0.001
Peri-I	229.81 ± 53.64	224.72 ± 54.80	212.03 ± 59.96	17.77 ± 31.45	35.879	< 0.001

ΔThickness, change in choroidal thickness between the baseline and the 2-year follow-up

*SER*= spherical equivalent of refraction; *D*= dioptre; *AL*= axial length; *RNFL*= retinal nerve fibre layer; *GCL + IPL*= ganglion cell layer and inner plexiform layer; *HFL + ONL*= Henle fibre layer and outer nuclear layer; *Para*= parafoveal; *Peri*= perifoveal; *N*= nasal; *T*= temporal; *S*= superior; *I*= inferior

^a,b^The same superscript letter indicate a significant difference between the two results after pairwise comparison using Bonferroni correction

^c^Medians (interquartile range) for SER and AL

^d^The means ± standard deviations for RNFL, GCL + IPL, HFL + ONL, retina, and choroid

**Fig. 2:**
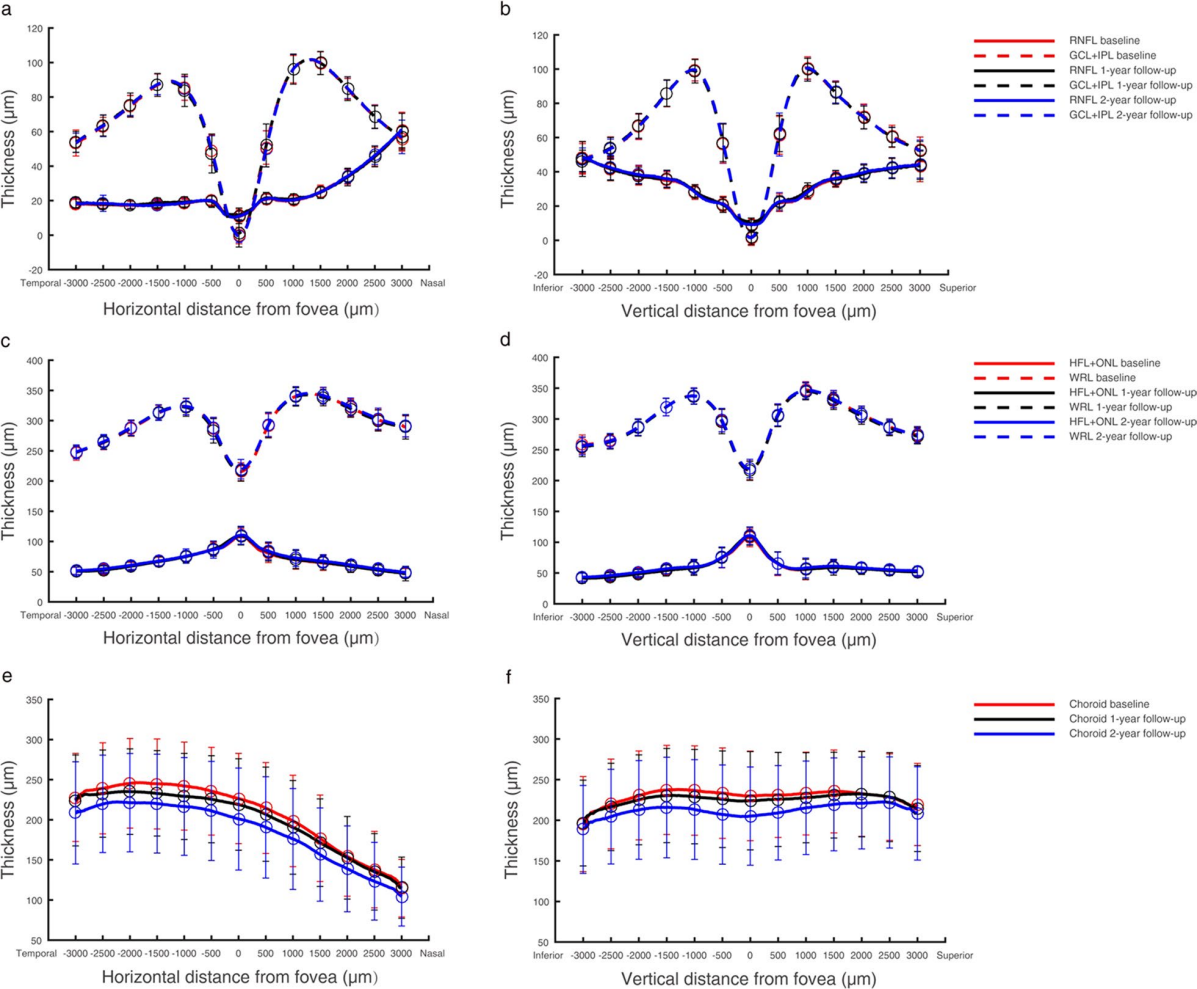
Thickness profiles of the retina and choroid in 168 myopic children. The thickness profiles of three intra-retinal layers, the total retina, and the choroid in horizontal and vertical scans were obtained by SD-OCT and averaged over each test. Thickness values for the RNFL, GCL + IPL, HFL + ONL, and WRL were similar; therefore, they overlapped each other in the figure and cannot be seen separately. **a**, **b** Horizontal (left) and vertical (right) thickness profiles of the RNFL and GCL + IPL; **c**, **d** horizontal (left) and vertical (right) thickness profiles of the HFL + ONL and whole retina; **e**, **f** horizontal (left) and vertical (right) thickness profiles of the choroid. RNFL, retinal nerve fibre layer; GCL + IPL, ganglion cell layer and inner plexiform layer; HFL + ONL, Henle fibre layer and outer nuclear layer; WRL, whole retinal layer

In the central macula, the baseline retinal thickness was 251.12 ± 15.91 µm, which at the 2-year follow-up was increased to 253.47 ± 15.74 µm. This retinal thickening was mainly due to the increased thickness of the HFL + ONL, from 88.29 ± 10.67 µm at baseline to 90.16 ± 10.49 µm at the 2-year follow-up (*P* < 0.001, Table 2, Fig. 2a–d).

The subfoveal CT at baseline was 231.03 ± 54.04 µm and became thinner at each follow-up visit, decreasing to 206.53 ± 59.71 µm in the second year (F = 73.358 *P* < 0.001). The same results were seen in the parafoveal nasal, temporal, superior, inferior, and perifoveal regions. During the 2-year study period, the minimum decrease in CT was 11.20 ± 38.41 µm in the superior perifovea, and the maximum decrease was 24.63 ± 31.16 µm in the central fovea (Table 2, Fig. 2e, f). Annual variation in CT demonstrated that the thinning amount in the second year was significantly greater than that in the first year (− 16.64 ± 25.63 µm vs. − 7.98 ± 23.61 µm, t = 2.941, *P* = 0.004).

### Factors associated with longitudinal changes in CT in the central fovea

Changes in subfoveal CT in the first year were significantly and independently correlated with sex, baseline values for age, AL, whole retinal thickness, and baseline subfoveal CT (Table 3). At the 2-year follow-up, more significant thinning of the central foveal choroid was associated with female sex (β = 17.258, *P* = 0.001), older age (β =  − 4.411, *P* = 0.049), thicker baseline subfoveal CT (β =  − 0.081, *P* = 0.046), and greater elongation of the AL (β =  − 43.579, *P* = 0.002). Univariate linear regression analysis revealed significant negative associations between the baseline subfoveal CT and baseline AL (slope =  − 17.88, r =  − 0.241, *P* = 0.002) values and between the 1-year and 2-year changes in the axial length and CT (slope =  − 51.79, r =  − 0.323, and *P* < 0.001 and slope =  − 58.06, r =  − 0.434, and *P* < 0.001, respectively).

**Table 3:** Multiple regression analysis of factors associated with 1- and 2-year longitudinal changes in choroidal thickness in the central fovea

Factor	Subfoveal choroidal thickness change (µm)^a^			
	One-year ∆thickness (95% CI)	*P*	Two-year ∆thickness (95% CI)	*P*
Age (years)	− 2.36 (− 5.74 to 1.01)	0.168	− 4.41 (− 8.81 to 0.01)	0.049
Sex	− 16.39 (− 24.13 to − 8.66)	< 0.001	− 17.25 (− 27.19 to − 7.32)	0.001
Lens	− 6.62 (− 14.44 to 1.18)	0.096	− 6.11 (− 16.02 to 3.79)	0.227
Baseline AL (mm)	− 8.87 (− 14.52 to − 3.21)	0.002	− 5.11 (− 12.30 to 2.07)	0.163
Baseline SER (D)	− 1.78 (− 7.66 to 4.09)	0.355	2.96 (− 4.48 to 10.40)	0.435
Baseline whole retinal thickness (µm)	− 0.25 (− 0.46 to 0.03)	0.024	− 0.19 (− 0.47 to 0.07)	0.158
Baseline subfoveal choroidal thickness (µm)	− 0.06 (− 0.12 to 0.01)	0.040	− 0.08 (− 0.16 to − 0.001)	0.046
One-year AL change (mm)	− 49.66 (− 79.68 to − 19.64)	0.001		
Two-year AL change (mm)			− 43.58 (− 71.41 to − 15.74)	0.002
One-year SER change (D)	4.86 (− 5.22 to 14.95)	0.345		
Two-year SER change (D)			9.08 (− 1.07 to 19.23)	0.080

*CI=* confidence interval; *AL=* axial length; *SER=* spherical equivalent of refraction

^a^∆Thickness changes are based on estimates determined by regression analysis; lens includes three groups of lenses worn by the participants: personalized progressive addition lenses (PPALs), fixed progressive addition lenses (FPALs), and single vision lenses (SVLs)

No significant difference in the baseline subfoveal CT value was found between the sexes (males, 225.59 ± 51.94 µm; females, 237.01 ± 55.97, *P* = 0.172). However, at the 2-year follow-up visit, choroidal thinning was more significant (*P* < 0.001) for females (− 33.38 ± 25.49 µm) than for males (− 16.42 ± 36.70 µm). There were no differences in baseline CT between the 7- to 9-year-old and 10- to 12-year-old participants (*P* = 0.700) or in the amount of thinning at the 2-year follow-up (*P* = 0.989, Additional file 1: Table S1).

### Longitudinal changes in thickness of the retina and choroid in children with rapid progression and stable progression myopia

**Fig. 3:**
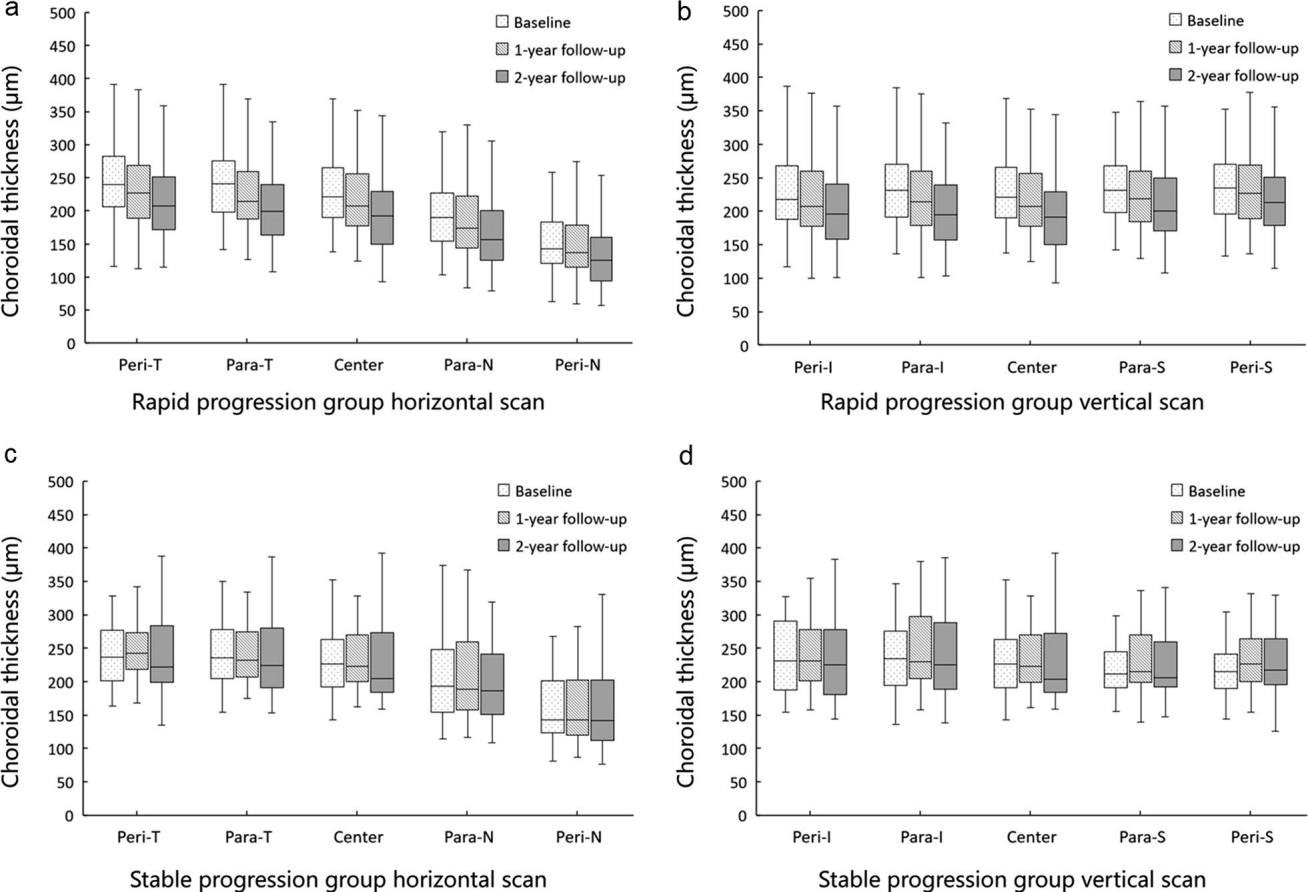
Longitudinal changes of fundic choroidal thickness in the central, parafoveal, and perifoveal regions of the rapid and stable progression groups. **a**, **b** Average choroidal thickness in horizontal and vertical scans of the rapid progression group. **c**, **d** Average choroidal thickness in horizontal and vertical scans of the stable progression group. Peri, perifoveal; Para, parafoveal; T, temporal; N, nasal; I, inferior; S, superior

Based on the changes in SER at the end of the 2-year study, we divided the children into two groups. For rapid progression, the SER increase was > 1.00 D (n = 129), and for stable progress, the SER increase was ≤ 1.00 D (n = 39). There were no significant differences in the baseline age, sex, SER, AL, or CT between the two groups (Additional file 1: Table S2). The AL in the two groups increased from 24.58 ± 0.73 mm and 24.55 ± 0.72 mm at baseline to 25.33 ± 0.75 mm (F = 1311.30, *P* < 0.001) and 24.97 ± 0.76 mm (F = 2227.26, *P* < 0.001) at the 2-year follow-up. The choroid in the rapid progression group continued to thin during the 2-year follow-up visits (F = 92.06, *P* < 0.001, Fig. 3). However, there was no significant difference in the change in CT during this 2-year study in the stable progression group (F = 2.23, *P* = 0.119).

## Discussion

Previous studies have shown that CT is correlated with refractive error and the AL and it is thinner in myopes than in hyperopes [25, 30]. However, the characteristics of retinal and choroidal thickness changes in myopic schoolchildren have not been clearly described. This longitudinal study included a large sample of myopic children to observe the morphological changes in retinal and choroidal thickness that correlated with changes in SER and AL over 2 years. There are three new important findings in this study. First, the macular CT of myopic children became thinner during the 2-year follow-up. Thinning was evident not only in the central fovea but also in the parafoveal and perifoveal regions. Second, thinning of the choroid occurred principally in the rapid progression group but not in the stable progression group. Third, the main influencing factors of CT in myopic children were axial growth and sex.

In this 2-year randomized controlled clinical study, personalized additional designed lenses did not produce a significant myopia control effect. This could indicate that the effect of near addition lenses on myopia control is limited [44, 45]. There was no significant difference in the change in CT between the three groups during this 2-year study. However, in general, the CT significantly decreased in the nine macular sectors of the Early Treatment Diabetic Retinopathy Study grid. Furthermore, CT was increasingly attenuated in the rapid progression group but not in the stable group, indicating that CT is continuously thinning in progressive myopia. This was consistent with the study conducted by Jin et al. [28], while not with the study by Xiong et al., who found a marked decrease in the CT for newly developed myopes but not in persistent myopes [29]. In European children with greater increases in AL, Hansen et al. [27] found that CT increased less or even became thinner. The varying outcomes can be attributed to factors such as different ethnicities, age, and refractive status. Taken together, we think that the thinning of CT and the decreasing choroidal blood perfusion, can cause scleral hypoxia, and may indicate the onset or faster progression of myopia.

Several studies have reported the correlation of CT with AL in children. In a cross-sectional study of 8- to 11-year-old myopic children, Qi et al. found that each millimetre of increase in AL was correlated with a reduction of 18.95 μm in subfoveal CT [25]. Additionally, Xiong et al. found that a 1-mm increase in AL was accompanied by a 26.66-μm decrease in the thickness of the choroid [29]. Our current study found that for each 1-mm increase in AL during the first year, CT decreased 51.79 μm, and for the second year, the decrease was 64.56 μm. Additionally, the progression of refractive error and increases in AL in our study were larger than those reported by others [28, 29]. The larger decreases in CT that we found can be attributed to the fact that this was the first 2-year longitudinal study focused on school-aged children with simple low-to-moderate myopia. In addition, we found that the change in CT during the first year was smaller compared to the second year, although the difference of AL change was not so obvious. We believe that the change of CT during the myopic progression was not entirely due to the passive stretch thinning caused by the expansion of the vitreous chamber. There is also other active mechanism which adjusts the input of visual information [46].

Two other factors that influenced CT were sex and age. Several studies have shown no difference in CT between male and female children [20, 47, 48], and our results are consistent. However, at the 2-year follow-up, the thinning of the choroid of female participants was significantly greater than that of the male participants. The specific reasons for this sex difference need to be further studied. The relationship between CT and age was confused by the different degrees of SER and the different myopia progression. Therefore, the results of previous studies are inconsistent. Xiong et al. confirmed that CT was positively correlated with age for emmetropes and mild myopes but not for children with SERs ≤ 2.00 D [26]. Our study found that there was no difference in CT changes between the two age groups. This may be related to our narrow age group (7 to 12 years old) and similar distribution of dioptres (of which 79.2% were ≤ 2.00 D).

There are some limitations to our study. First, this study was a part of PACT [31]. Even though the results showed no significant differences in the SER, AL, and CT values among three groups (two treatment groups and one control group), our data cannot completely rule out the effect of different myopia control lenses on CT. The second limitation is that we did not analyse the role of growth and systemic development (i.e., changes in body height, weight, and puberty), in the changes in CT. The third was that we only observed myopia progression for 2 years.

## Conclusions

The macular CT of myopic children, especially those with rapid progression myopia, was significantly attenuated at the 1- and 2-year follow-up visits. Therefore, it suggests an association between CT thinning and myopia progression and axial elongation. The control of CT may play an important role in the regulation of children ocular growth.

## Supplementary Information

**Additional file 1: Table S1.** Longitudinal changes in ocular biological parameters stratified by sex and age. **Table S2.** Comparison of baseline data between the rapid progression and stable progression groups.

## Data Availability

The datasets used and analysed in our study are available upon reasonable request from the corresponding author.
