# Supplementary material for: Two-year longitudinal change in choroidal and retinal thickness in school-aged myopic children: exploratory analysis of clinical trials for myopia progression
Source: Eye Vis (Lond). 2022 Feb 1;9:5. doi: 10.1186/s40662-022-00276-4 (PMC8805434; doi:10.1186/s40662-022-00276-4)
Supplement: Supplementary file 1 — Additional file 1: Table S1. Longitudinal changes in ocular biological parameters stratified by sex and age. Table S2. Comparison of baseline data between the rapid progression and stable progression groups. [file 40662_2022_276_MOESM1_ESM.docx]

**Table S1.** Longitudinal changes in ocular biological parameters stratified by sex and age.

| Parameter | | Males  (n=88) | Females  (n=80) | *P* | 7–9 years  (n=101) | 10–12 years  (n=67) | *P* |
| --- | --- | --- | --- | --- | --- | --- | --- |
| AL (mm) |  |  |  |  |  |  |  |
| Baseline | | 24.87 ± 0.70 | 24.25 ± 0.61 | < 0.001 | 24.45 ± 0.69 | 24.76 ± 0.74 | 0.007 |
| One-year follow-up | | 25.23 ± 0.74 | 24.63 ± 0.61 | < 0.001 | 24.86 ± 0.74 | 25.07 ± 0.73 | 0.070 |
| Two-year follow-up | | 25.52 ± 0.78 | 24.94 ± 0.62 | < 0.001 | 25.18 ± 0.77 | 25.34 ± 0.74 | 0.170 |
| Change | | 0.64 ± 0.25 | 0.68 ± 0.22 | 0.301 | 0.72 ± 0.27 | 0.58 ± 0.17 | < 0.001 |
| CT (µm) |  |  |  |  |  |  |  |
| Baseline | | 225.59 ± 51.94 | 237.01 ± 55.97 | 0.172 | 232.34 ± 51.22 | 229.05 ± 58.37 | 0.700 |
| One-year follow-up | | 222.82 ± 56.64 | 224.69 ± 57.24 | 0.832 | 224.96 ± 55.28 | 221.83 ± 59.30 | 0.728 |
| Two-year follow-up | | 209.16 ± 62.28 | 203.62 ± 56.99 | 0.550 | 207.87 ± 58.35 | 204.50 ± 62.09 | 0.722 |
| Change | | −16.42 ± 36.70 | −33.38 ± 25.49 | 0.001 | −24.47 ± 33.49 | −24.54 ± 32.19 | 0.989 |

AL= axial length; CT= choroidal thickness

**Table S2.** Comparison of baseline data between the rapid progression and stable progression groups.

| Parameter^*,†,^ | Rapid progression  (n=129) | Stable progression  (n=39) | t | *P* |
| --- | --- | --- | --- | --- |
| Age (years) | 9.20 ± 1.04 | 9.44 ± 1.14 | −1.198 | 0.233 |
| Sex (male, %) | 63 (48.8%) | 25 (64.1%) | 2.798 | 0.103 |
| SER (D) | −2.44 ± 0.61 | −2.22 ± 0.61 | −1.970 | 0.053 |
| AL (mm) | 24.58 ± 0.73 | 24.55 ± 0.72 | 0.262 | 0.795 |
| CT (µm) | 231.80 ± 56.24 | 228.48 ± 46.61 | 0.335 | 0.738 |
| SER, spherical equivalent of refraction; D, dioptre; AL, axial length; CT, choroidal thickness  *, Values for age, SER, AL, and CT are the means ± standard deviations; ^†^, Values for sex are the median (interquartile range) | | | | |
